# Overproduction of the Flv3B flavodiiron, enhances the photobiological hydrogen production by the nitrogen-fixing cyanobacterium *Nostoc* PCC 7120

**DOI:** 10.1186/s12934-020-01320-5

**Published:** 2020-03-10

**Authors:** Baptiste Roumezi, Luisana Avilan, Véronique Risoul, Myriam Brugna, Sophie Rabouille, Amel Latifi

**Affiliations:** 1 Aix Marseille Univ, CNRS, LCB, Laboratoire de Chimie Bactérienne, Marseille, France; 2 Aix Marseille Univ, CNRS, BIP, Laboratoire de Bioénergétique et Ingénierie des Protéines, Marseille, France; 3 Sorbonne Université, CNRS, Laboratoire d’Océanographie de Villefanche, LOV, 06230 Villefranche-sur-Mer, France; 4 Sorbonne Université, CNRS, Laboratoire d’Océanographie Microbienne, LOMIC, 66650 Banyuls-sur-Mer, France

**Keywords:** Cyanobacteria, Flavodiiron, Heterocyte, Hydrogen, Hydrogenase

## Abstract

**Background:**

The ability of some photosynthetic microorganisms, particularly cyanobacteria and microalgae, to produce hydrogen (H_2_) is a promising alternative for renewable, clean-energy production. However, the most recent, related studies point out that much improvement is needed for sustainable cyanobacterial-based H_2_ production to become economically viable. In this study, we investigated the impact of induced O_2_-consumption on H_2_ photoproduction yields in the heterocyte-forming, N_2_-fixing cyanobacterium *Nostoc* PCC7120.

**Results:**

The *flv3B* gene, encoding a flavodiiron protein naturally expressed in *Nostoc* heterocytes, was overexpressed. Under aerobic and phototrophic growth conditions, the recombinant strain displayed a significantly higher H_2_ production than the wild type. Nitrogenase activity assays indicated that *flv3B* overexpression did not enhance the nitrogen fixation rates. Interestingly, the transcription of the *hox* genes, encoding the NiFe Hox hydrogenase, was significantly elevated, as shown by the quantitative RT-PCR analyses.

**Conclusion:**

We conclude that the overproduced Flv3B protein might have enhanced O_2_-consumption, thus creating conditions inducing *hox* genes and facilitating H_2_ production. The present study clearly demonstrates the potential to use metabolic engineered cyanobacteria for photosynthesis driven H_2_ production.

## Background

Development of renewable fuel as a clean alternative to fossil fuels is nowadays strongly needed. Besides solar energy, which represents the most abundant renewable energy, hydrogen (H_2_) is regarded as an attractive option for its high energy content and null ecological impact: its combustion only releases water as a byproduct. In this regard, growing autotrophic, photosynthetic organisms (cyanobacteria and algae) to yield H_2_ with minimized energy supply is a very promising alternative to fossil fuels.

In cyanobacteria, H_2_ is produced by two different enzymes: hydrogenase and nitrogenase. In diazotrophic strains, H_2_ is formed as a by-product of N_2_ fixation activity performed by the nitrogenase. However, the nitrogenase is often associated to an uptake hydrogenase, encoded by the *hup* genes that catalyze the oxidation of H_2_ into protons; the amount of H_2_ produced during nitrogen fixation is thus rather limited [1]. The second type of enzymes producing H_2_ are hydrogenases (H_2_ases). Bidirectional NiFe H_2_ases (called Hox), which catalyze both H_2_ oxidation and proton reduction, are largely distributed across the cyanobacterial phylum [2, 3]. They form a heteropentamer with a H_2_ase part (HoxYH) and a diaphorase part (HoxEFU). The physiological function of Hox hydrogenases in cyanobacteria is not well understood but they may serve as electron valve during photosynthesis in the unicellular cyanobacterium *Synechocystis* sp. PCC 6803 [4]. The expression of *hox* genes is induced in dark and/or anaerobic conditions [5] and is under the control of the regulators LexA and two members of the AbrB family (antibiotic resistance protein B) [6–8]. The sensitivity of cyanobacterial bidirectional H_2_ases to oxygen (O_2_) and the fact that their activity occurs in the dark or under anaerobic conditions are the major obstacles to obtaining efficient solar driven production of H_2_ in cyanobacteria. Several strategies have so far been adopted to overcome the limits of the natural H_2_-evolving mechanisms in cyanobacteria (for a review see [9]).

During photosynthesis, O_2_ can be reduced to water through an enzymatic process involving flavodiiron proteins (Flvs) [10]. In cyanobacteria, Flvs catalyze the reduction of O_2_ into water using NADPH as an electron donor [11] and play a critical role during growth under fluctuating light regimes [12]. The filamentous heterocyte-forming cyanobacterium *Anabaena*/*Nostoc* PCC7120 (hereafter *Nostoc*) produces four Flvs proteins in the vegetative cells (Flv1A, Flv2, Flv3A, and Flv4) and two Flvs (Flv1B and Flv3B) specific to the heterocyte [13]. The Flv3B protein mediates light-induced O_2_-uptake in the heterocyte, which benefits nitrogenase activity by providing a protection mechanism against oxidation [14]. In addition, the *∆flv3B* mutant displayed a broad effect on gene expression, which indicates that a regulation process links gene transcription to O_2_ level in the heterocyte [14].

We recently reported that decreasing the O_2_ level inside the heterocyte by producing the cyanoglobin GlbN allowed it to host an active FeFe H_2_ase from *Clostridium acetobutylicum.* The recombinant strain displayed a significant H_2_-production yield under phototrophic conditions [15]. These data suggest that engineering approaches increasing the anaerobiosis inside the heterocyte can be highly profitable for the activity of O_2_-sensitive enzymes. To go further, we investigate here the impact of an overproduction of the flavodiiron Flv3B protein on the production of H_2_ in *Nostoc*. We demonstrate that the recombinant strain produces on average tenfold more H_2_ than the parental strain and that the expression of the *hox* genes is induced in this genetic background.

## Results

### Construction and characterization of a *Nostoc* recombinant strain overexpressing the *flv3B* gene

**Fig. 1:**
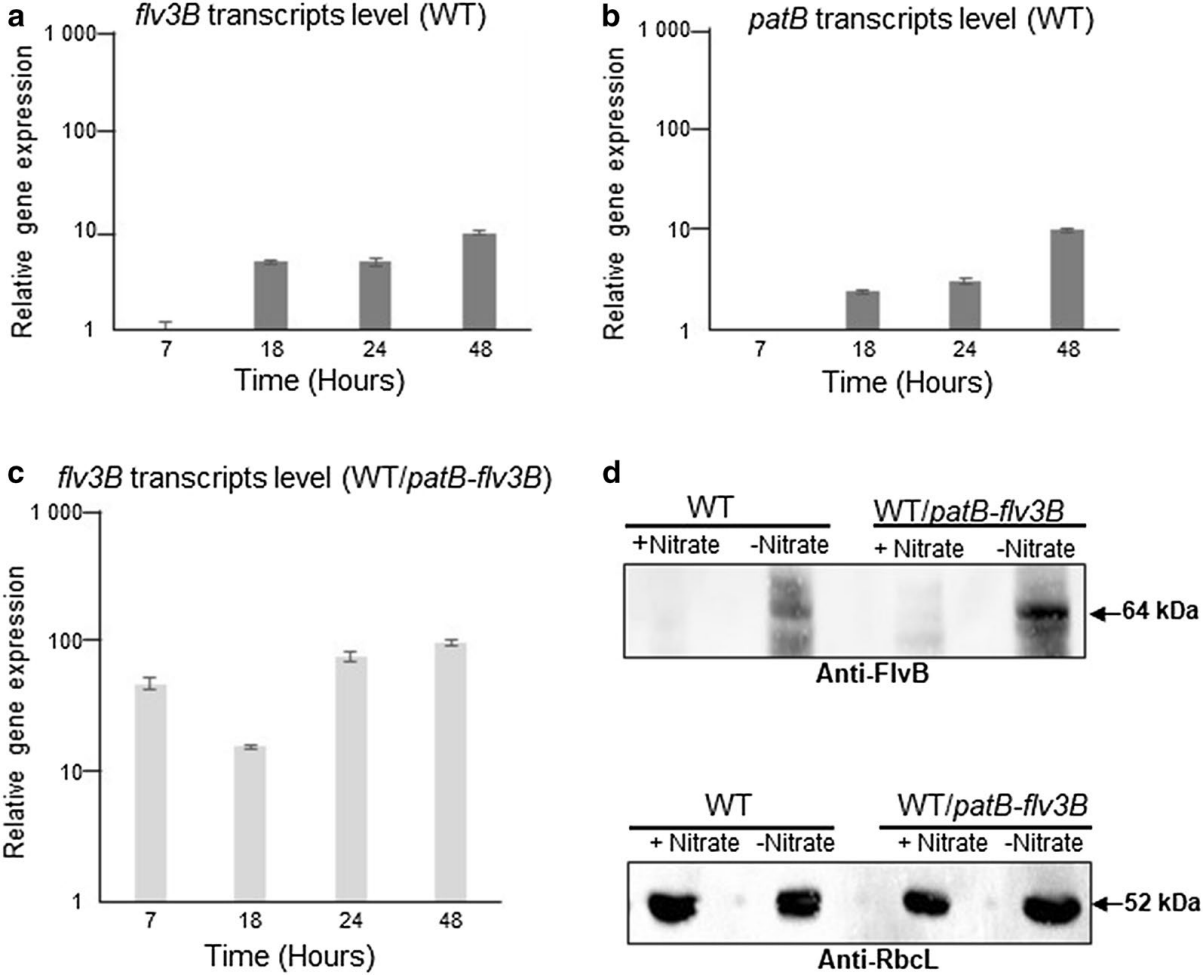
Flv3B overproduction analysis. **a**–**c** Quantitative RT-PCR analysis of *flv3B* (**a**, **c**) and *patB* (**b**, **d**) gene transcription. RNA were collected from the wild type (**a**, **b**) or the WT*/patB*-*flv3B* (**c**) strain at four different times (7, 18, 24 and 48 h) after the onset of nitrogen depletion. Each sample was measured in triplicate and the standard deviation is indicated by error bars. Values were normalized to the *rnpB* transcript, relatively to the value obtained for the wild type strain, which was set to 1. **d** Immunoblot analysis of the amount of Flv3B protein (upper panel) in the wild type and WT/*patB*-*flv3B* strains, carried out using antibodies produced against FlvB from *Chlamydomonas reinhardtii* [18]. Immunoanalysis of RbcL protein amount was carried out as a loading control (lower panel). The condition (+ Nitrate) stands for cultures performed in nitrate-containing medium, and the condition (− Nitrate) indicates cultures grown in nitrate-free medium

**Fig. 2:**
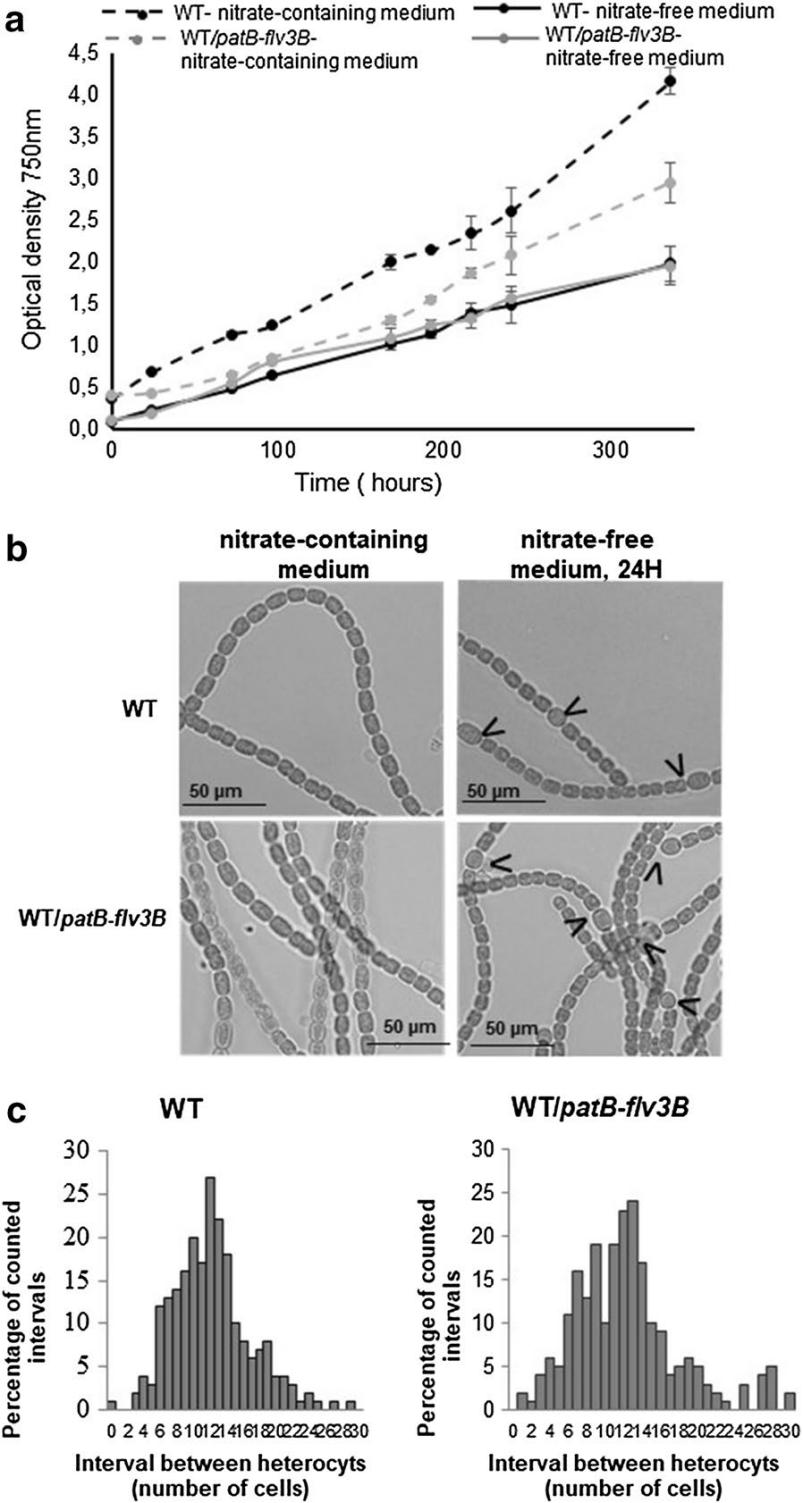
Characterization of *Nostoc* strain overexpressing the *flv3B* gene. **a** Growth curve of *Nostoc* strains grown in either nitrate-containing medium or nitrate free medium. For each curve, three independent cultures were performed. The growth was assessed during 12 days by measuring the optical density at 750 nm. The standard deviation is indicated by error bars. **b** Light microscope images of *Nostoc* strains grown in nitrate-containing medium or nitrate- free medium. For the last conditions, images were acquired 24 h after nitrogen starvation. Heterocytes are indicated by black arrows. **c** Heterocyte pattern formation in the wild type and the WT*/patB*-*flv3B* strain. Strains were grown in BG11 (nitrate-containing medium) to an OD_750_ of 0.4 and induced to form heterocytes by transfer to BG-110 medium (nitrate-free medium). Vegetative cells and heterocytes were scored microscopically 24 h after nitrogen starvation. The data shown are representative of three independent experiments

In a transcriptomic study using an RNAseq approach, the transcription of *flv3B* (all0178) gene was induced 12 h after nitrogen starvation [16]. In order to specifically overexpress the *flv3B* gene in the heterocytes without competing with the natural promoter of this gene, we decided to place it under the control of a heterocyte-specific promoter whose transcription is induced at the same time than *flv3B*. For this, we analyzed the transcription of *flv3B* throughout the differentiation process by quantitative RT-PCR. We also concomitantly monitored the transcription of the *patB* gene, known to be expressed after the initiation of heterocytes development [17]. *flv3B* and *patB* genes showed very similar transcription profile (Fig. 1). Both genes were induced 18 h after nitrogen stepdown and their transcription increased through the development program (compare Fig. 1a, b). The *patB* promoter was therefore chosen to drive *flv3B* overexpression in *Nostoc*, and the resultant recombinant strain was named WT*/patB*-*flv3B.* As a first step in the characterization of this strain, we checked the overexpression of *flv3B* in response to nitrogen starvation. We first carried out quantitative RT-PCR analyses and expressed the amount of *flv3B* transcripts in the recombinant strain relatively to their amount in the wild type. Results reveal a more than tenfold increase in *flv3B* gene expression in the recombinant strain, also starting much sooner after nitrate depletion, indicating that *flv3B* gene was strongly overexpressed (Fig. 1c). Because Flv3B from *Nostoc* and FlvB from *Chlamydomonas reinhardtii* amino acid sequences present 51% identity (Additional file 1: Figure S1), we hypothesized that antibodies produced against FlvB from *C. reinhardtii* [18] could cross-react with Flv3B and hence could be used to analyze the amount of Flv3B protein in *Nostoc*. Since Flv1B from *Nostoc* displays 30% identity with FlvB from *C. reinhardtii*, the anti-FlvB antibodies could also cross-react with this protein. However, as only the *flv3B* gene was overexpressed, we assumed that FlvB antibodies could help assessing Flv3B overproduction. In the western blot analyses, the amount of RbcL protein served to check that equal amounts of proteins were loaded in each condition [19]. Data on Fig. 1d show that a protein of the expected size of Flv3B (64 kDa) was detected only in BG11_0_ medium (without nitrate), which is in agreement with *flv3B* gene being specific to the heterocyte [13]. Moreover, this protein accumulated at a higher level in the WT*/patB*-*flv3B* strain. Altogether, these results indicate that the *flv3B* gene was overexpressed in the recombinant strain. The WT*/patB*-*flv3B* strain showed similar growth efficiency than the wild type under both nitrogen replete and deplete conditions (Fig. 2a, Table 1), and both strains differentiated heterocytes equally well (Fig. 2b). The frequency of heterocytes along the filament was similar between the two strains, with 12 vegetative cells on average in between two heterocytes (Fig. 2c). Given that the overexpression of *flv3B* did not impair the growth ability of the strain, we proceeded with an analysis of its impact on H_2_-production.

**Table 1:** Nitrogenase activity of the cyanobacterial strains studied

Strain measure	Wild type	WT*/patB*-*flv3B*
Exponential growth rate per day (BG110)	0.155	0.155
Chl*a* content (mg Chl*a*/mL)	4.49	8.9
Nitrogenase activity (nmol N_2_/mg Chl*a*/h)	17.3	11.2
sd on nitrogenase activity	0.001	0.00,025

Two independent cultures of each strain were grown as explained in the Materials and methods section. For each strain, the nitrogenase activity values presented in this table were registered at T = 4 h of the light phase

*Chla* chlorophyll *a*, *sd* standard deviation

### *flv3B* overexpression in the heterocyte powers H_2_-production

**Fig. 3:**
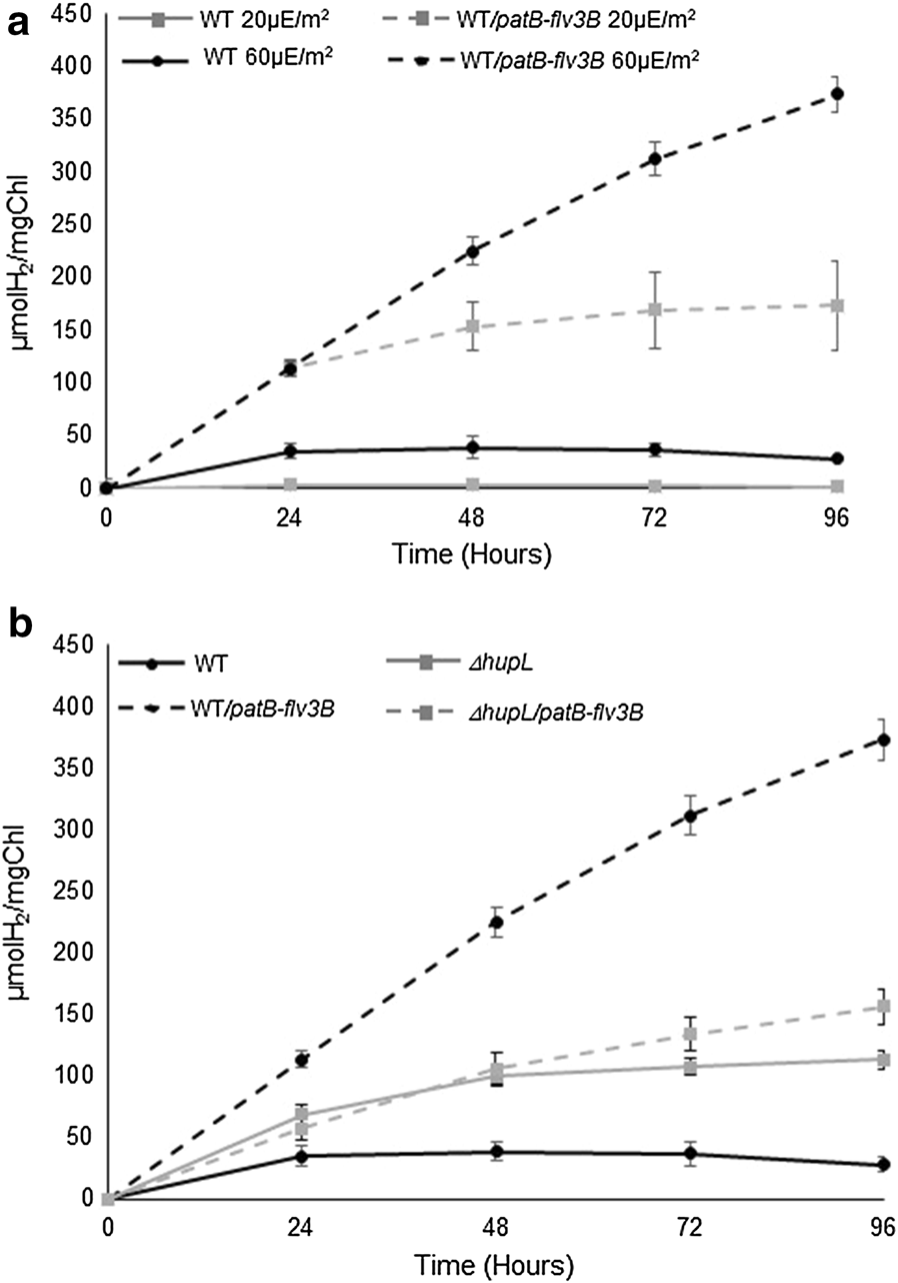
H_2_ production kinetics. **a** Wild type or WT/*patB*-*fvl3B* were grown in nitrate-containing medium until OD 750 nmm = 0.8. Heterocyte formation was induced by transferring the strains to a nitrate-free medium during 24 h. The strains were then incubated under light intensities of either 20 µE/m^2^ or 60 µE/m^2^, and H_2_ production was assessed by chromatography as explained in the methods section during 4 days. The values represent Mean ± SEM (n = 8). **b** Wild type, WT/*patB*-*fvl3B, ∆hupL or ∆hupL/patB*-*fvl3B* strains were grown under light intensities of 60 µE/m^2^. Hetrocyte formation and H_2_-production were respectively induced and performed as described above. The values represent Mean ± SEM (n = 8)

The sensitivity of H_2_ases and nitrogenase to O_2_ is an important limitation to H_2_-photoproduction. By promoting O_2_ consumption in the heterocyte, the Flv3B protein is ought to protect enzymes evolving H_2_. To test this hypothesis, the wild type and the WT*/patB*-*flv3B* strains were first grown exponentially under aerobic conditions in nitrate replete medium. H_2_-production yield was then measured and compared after cells were transferred to nitrate-depleted medium. The recombinant strain produced 10 to 30-fold more H_2_ than the wild type under the same conditions (Fig. 3a). H_2_ production increased with the experienced light irradiance, with the highest yield obtained under 60 µE m^−2^. Flv3B overproduction is thus an efficient way to enhance H_2_ photoproduction in *Nostoc*.

### The presence of the uptake H_2_ase is required for a maximal H_2_ production

Since the uptake H_2_ase consumes the H_2_ produced by the nitrogenase in the heterocyte and since its deletion enhanced H_2_ production [20], we investigated whether a deletion of *hupL* gene, encoding the large subunit of the uptake H_2_ase would show a cumulative effect with Flv3B overproduction. For this purpose, a deletion of *hupL* was constructed and the resultant strain transformed with the *patB*-*flv3B* containing plasmid. The deletion of *hupL* gene in an otherwise wild type background increased the H_2_ production level, which is in agreement with data published previously [20] (Fig. 3b). However, the absence of a further enhanced H_2_ production following the overproduction of Flv3B in the ∆*hupL* strain was unexpected. Intriguingly, the ∆*hupL/patB*-*flv3B* strain produced 3.5-fold less H_2_ than the *WT/patB*-*flv3B* strain (Fig. 3b).

### Flv3B overproduction does not stimulate nitrogenase activity

The deletion of the *flv3B* gene was shown to result in a decrease in both the amount of nitrogenase subunits and nitrogenase activity [14]. Therefore, the increased H_2_ production in the *flv3B* overproducing strain could be a consequence of an increase in the activity of the nitrogenase. To test this hypothesis, we monitored nitrogenase activity in exponentially growing cultures after their transfer to a medium devoid of combined nitrogen. Results demonstrated that the overproduction of Flv3B protein did not enhance nitrogenase activity (Table 1). Therefore, the effect of Flv3B on H_2_ production is unlikely to result from nitrogenase activity.

### Flv3B overproduction induces the expression of the bidirectional H_2_ase encoding genes

**Fig. 4:**
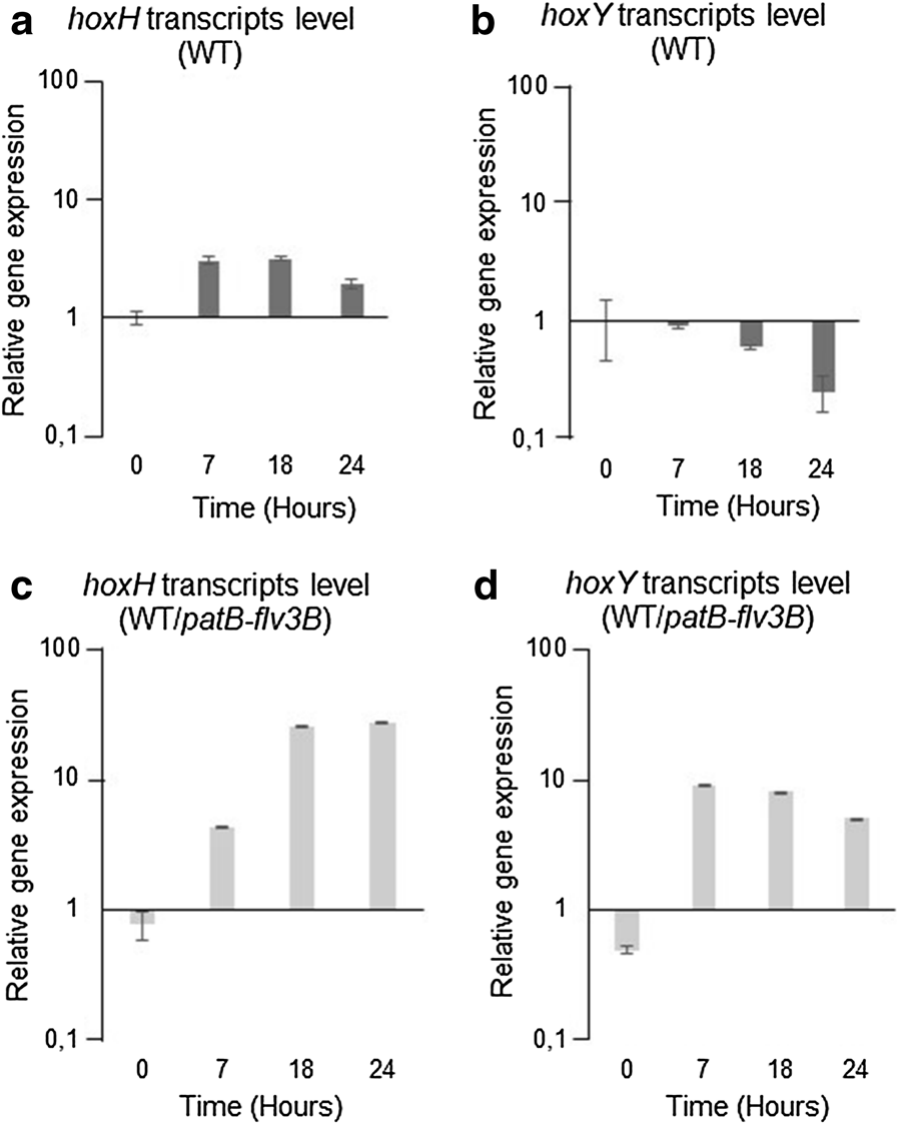
*hoxY, H* genes transcription analysis. Quantitative RT-PCR analysis of *hoxY* and *hoxH* gene transcription. RNA were collected form wild type (**a**, **b**) or WT/*patB*-*fvl3B* (**c**, **d**) at different times after the onset of the nitrogen depletion step. Each sample was measured in triplicate and the standard deviation is indicated by error bars. Values were normalized to the *rnpB* transcript

**Fig. 5:**
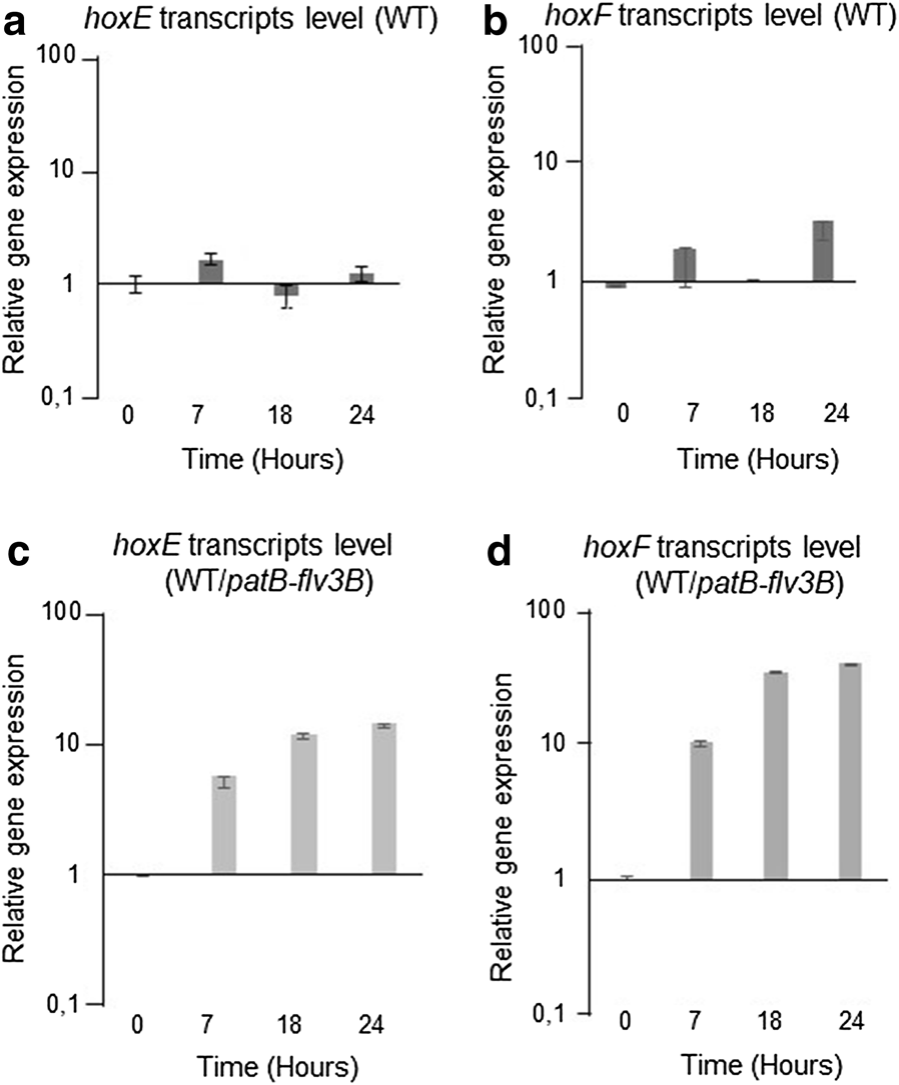
*hoxE, F* genes transcription analysis. Quantitative RT-PCR analysis of *hoxE* and *hoxF* gene transcription. RNA were collected form wild type (**a**, **b**) or WT/*patB*-*fvl3B* (**c**, **d**) at different times after the onset of the nitrogen depletion step. Each sample was measured in triplicate and the standard deviation is indicated by error bars. Values were normalized to the *rnpB* transcript

Since the only other enzyme able to produce H_2_ in cyanobacteria is the bidirectional Hox H_2_ase, we analyzed whether an induced expression of *hox* genes then results from the overproduction of Flv3B. The *hoxH* and *hoxY* genes encoding the H_2_ase subunits as well as the *hoxE,F,U* genes encoding the diaphorase subunits belong to two separate operons [21]. To evaluate the expression of these operons, the transcription of two genes from each operon (*hoxH,Y* and *hoxE,F*) was comparatively monitored in the wild type and the recombinant strains. Quantitative RT-PCR analysis was used to evaluate the transcription of these four genes after transfer of the strains into nitrogen deplete conditions to induce *flv3B* expression. The transcription of the four *hox* genes was weak in the wild type strain (Figs. 4a, b;  5a, b), which is in agreement with the fact that the *hox* genes are not expressed under aerobic conditions [21]. However, in the WT/*patB*-*flv3B* strain, 18 h after nitrogen step down, the *hoxE,F, H and Y* transcripts level were on average tenfold higher than in the wild type (Figs. 4c, d and 5c, d). The expression of the two *hox* operons encoding the H_2_ase and diaphorase proteins is therefore induced in the strain overexpressing the *flv3B* gene under the heterocyte specific promoter *patB*. Consequently, the effect of *flv3B* overexpression on H_2_ production may be mediated by the induction of *hox* genes.

## Discussion

In this work we show that overexpression of *flv3B* gene from a promoter specific to the heterocyte enhanced the production of H_2_ in aerobic cultures of *Nostoc*. So far, the only conditions in which H_2_-evolution had been recorded in aerobically grown *Nostoc* were the use of mutants lacking the HupL subunit of the uptake H_2_ase or the last step of the maturation system of this H_2_ase [20, 22]. H_2_ evolution mediated by Flv3B overproduction presents the advantage of sustaining the protective effect of the uptake H_2_ase on the nitrogenase.

By studying the phenotype of a *∆flv3B* mutant of *Nostoc*, Ermakova et al. [14] showed that Flv3B protected nitrogenase through light-induced O_2_ consumption inside the heterocytes. The effect of Flv3B overproduction evidenced in our work could therefore result from a stimulated nitrogenase activity. But the recombinant strain displayed similar nitrogenase activity as the wild type (Table 1), proof that another mechanism operates to enhance H_2_ production.

In *C. reinhardtii,* the existence of intracellular microoxic niches in the chloroplast preserve FeFe-hydrogenase activity and support continuous H_2_ production during growth in aerobic cultures [23]. The same authors suggested that Flvs proteins were involved in this process [23]. A similar mechanism may be proposed to explain the effect of the Flv3B protein overproduction on H_2_ evolution, in which the decrease in O_2_ concentration in the heterocyte would reinforce the anaerobiosis in this cell type, thus promoting H_2_ase synthesis and/or activity. We studied the transcription of *hox* genes encoding the bidirectional H_2_ase as their induction is known to be concomitant to high H_2_ase activity [21]. Data in Figs. 4, 5 indicate that *flv3B* overproduction led to a substantial induction of *hoxE,F,H,Y* genes expression that can explain the H_2_ production measured in this strain. The LexA transcriptional factor was proposed to regulate *hox* genes transcription in *Nostoc* [21]. In the unicellular cyanobacterium *Synechocystis* PCC6803, LexA was suggested to act as a transducer of the intracellular redox state, rather than of the SOS response as in *E. coli* [24]. Based on this information, we suggest that an increased O_2_-uptake driven by Flv3B overproduction can modify the redox state in the heterocytes, resulting in the observed induction of *hox* genes transcription.

**Fig. 6:**
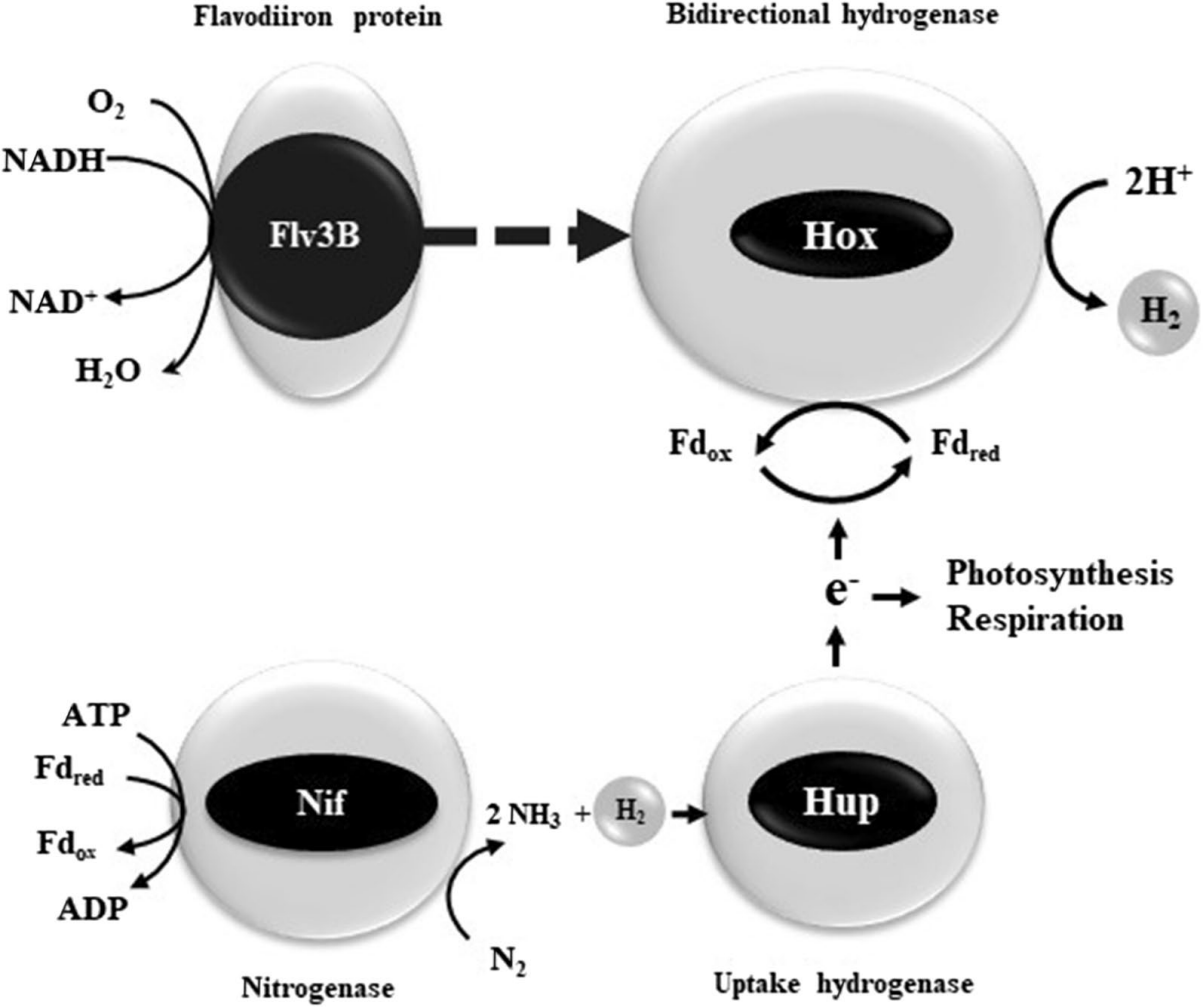
Hypothetical model of H_2_ production in *Nostoc* strain overproducing Flv3B. Nitrogen fixation occurring in the heterocyte produces H_2_ which is recycled by the Hup H_2_ase. Overexpression of the *flv3B* gene increases the uptake of O_2_ reinforcing the microoxie inside the heterocyte. The induction of *hox* genes transcription leads to H_2_ production. Fd_red_: reduced ferredoxin; Fd_ox_: oxidized ferredoxin. Dashed lines stand for indirect effect

Surprisingly, and contrary to what happens in the wild type background, the lack of the uptake H_2_ase in the WT/*patB*-*flv3B* strain led to a decrease in H_2_ production (Fig. 3b). As the H_2_ases are bidirectional enzymes, a possible interpretation of this result is that the Hup enzyme is responsible of the H_2_ production observed in this recombinant strain. However, this is rather unlikely since it was demonstrated that the Hup H_2_ase is not able to produce H_2_ at any significant rate, and is considered to react only in the uptake direction [1, 25]. Through the oxidation of H_2_, the Hup H_2_ase provides electrons to the photosynthesis and respiratory processes [1] (Fig. 6). Since the Hox H_2_ase was suggested to use ferredoxin as reducing partner rather than NAD(P)H as previously admitted (reviewed in [9]), this enzyme may benefit from the electrons generated by the Hup H_2_ase through regeneration of the reduced ferredoxin pool (Fig. 6). This could explain the negative impact of the *hupL* deletion on the H_2_-production yield in the WT/*patB*-*flv3B* strain (Fig. 6). Our data show that metabolic engineering approaches are particularly relevant in the use of photosynthetic bacteria for biofuel production.

## Conclusion

In the present study, the *flv3B* gene was specifically overexpressed in the heterocyte of *Nostoc* under the control of the *patB* promoter. The overproduction of the Flv3B flavodiiron enhanced the H_2_ production yield by a factor of ten on average, which is not to be attributed to the nitrogenase since no increase in the nitrogenase activity was observed. The transcription of the *hox* genes was induced in the recombinant strain expressing the *flv3B* gene, suggesting that the additional H_2_ produced relates to the activity of the bidirectional H_2_ase. Facilitating the consumption of O_2_ inside the heterocyte thus appears as a relevant step towards the design of an optimized *Nostoc* strain for H_2_ production. This paves the way to further improvement to achieve sustainable production of H_2_ by air-grown cyanobacteria.

## Methods

### Growth conditions and heterocytes induction

Cyanobacterial strains were grown in BG11 medium (nitrate replete) at 30 ℃ under continuous illumination (30 µE m^−2^ s^−1^). Cultures of recombinant strains were supplemented with neomycin (50 μg mL^−1^). Heterocyte formation was induced by transferring the exponentially growing cultures (OD 750 = 0.8) to BG11_0_ (BG11 devoid of sodium nitrate) by filtration (0.2 µm pore size filters, Sigma) and resuspension of cells into the nitrate-free medium. The growth was maintained for 4 days. The presence of heterocytes was confirmed by light microscopy and their distribution within filaments was rated visually by counting the number of vegetative cells between two heterocytes. At least 400 total vegetative cells were counted for each strain.

In the H_2_ production experiments, the strains were grown under continuous illumination of 20 µE m^−2^ s^−1^ or 60 µE m^−2^ s^−1.^

### Construction of plasmids and strains

To construct the Flv3B overproducing strain, the promoter region of *patB* (all2512, 500 bp upstream the start codon) was amplified by PCR from *Nostoc* sp. PCC 7120 genomic DNA using the *ppatB* forward and *ppatB* reverse primers (Table 2). The *ppatB* reverse primer contained a multiple cloning site (ApaI, ClaI, BamHI, SalI, ScaI, EcoRI). The amplified promoter was cloned into BglII and EcoRI restriction sites of the pRL25T plasmid [26], yielding the pRL25T-*patB* plasmid. The open reading frame of *flv3B* gene was amplified using the *flv3B* forward and reverse primers (Table 2), and cloned into the ApaI and ScaI restriction sites of the pRL*patB*. The recombinant plasmid (pRL25T-*patB*-*flv3B*) was analyzed by sequencing (Millegen). Conjugation of *Nostoc* was performed as described in Ref. [27]. Briefly, *E. coli* strains (bearing the replicative pRL25T-*patB*-*flv3B* and the RP-4 conjugative plasmid) grown to exponential growth phase, were mixed to an exponentially grown *Nostoc* culture. The mixture was plated on BG11 plates and Neomycin was added 24 h later for plasmid selection. Plasmid extraction was used to analyze the obtained recombinant clones.

**Table 2:** sequence of the primers used in this study

Name	Sequence (5′–3′)	Experiment
*rnpB* forward	TCGTGAGGATAGTGCCACAG	Quantitative RT-PCR analysis
*rnpB* reverse	GGAAGTTTCTTCCCCAGTCC	
*flv3B* RT forward	TTTGGTGGAAGATGTGCTGC	
*flv3B* RT reverse	GCCAATGTAAGTTAGGCGCA	
*patB* forward	AGGGGCGATGTAAAGTGGAA	
*patB* reverse	TTGACTGCTCGACTGTAGCA	
*hoxE* forward	GCGTCACCAGTATCAGCAAG	
*hoxE* reverse	TGGGGCGCTAGGGAAAATAA	
*hoxF* forward	ACCCGGCTGAATCTGGTTTA	
*hoxF* reverse	AAGCCTGTGTTGCGGATTTT	
*hoxH* forward	CTGGACAGGTAAACGATGCG	
*hoxH* reverse	ACAAATCCGCGCTGTAATCC	
*hoxY* forward	TTTCCTTTGGTGACTGTGCG	
*hoxY* reverse	GGTTGATATCGGCTGCTTGG	
*ppatB* forward	TATAAGATCTGTCTTTAAATATACATGGTTTGGG	Cloning of *patB* promoter
*ppatB* reverse	TATAGAATTCGAGCTCGTCGACCCGGGATCCATCGATGGGCCCCATATAACTTTCTTCCCACCC	
*flv3B forward*	TAT CCCGGG ATG GTA TCG ATG TCT ACG ACC	
*flv3B reverse*	TAT AGTACT TTA GTA ATA GTT GCC TAC TTT GCG	
Strp forward	AATTCCCCTGCTCGCGCAGG	Construction of the *hupL* deleted mutant
Strp reverse	AGCTTAGTAAAGCCCTCGCT	
all0678 forward	TTCGATATCTAGATCTCGAGTCAATTAATGACTTTTGACTAATTA	
all0678 reverse	AGTAGACGGAGTATACTAGTGCAACTTTCGGAGCG	
Strp-all0678 forward	CCTGCGCGAGCAGGGGAATTCATATAACTGCTGTGGCA	
Strp-all0678 reverse	AGCGAGGGCTTTACTAAGCTGTTTAAACGCAGAGGGG	

Deletion of the *hupL* gene, yielding the *∆hetL* strain, was obtained by homologous recombination replacing the *hupL*3′ gene (all0687C) with the gene encoding the spectinomycin/streptomycin resistance (Sp/Sm cassette hereafter). For this purpose, the upstream and downstream 1500 bp flanking the *hupL*3′ gene were amplified form *Nostoc* genomic DNA using the all0678 forward/all0678 reverse and the Strp-all0678 forward/Strp-all0678 forward, respectively; The Sp/Sm cassette was amplified using the Strp forward/Strp reverse primers (Table 2), using the pBAD42 plasmid (Addgen) as template. Gibson’s assembly technique (New-England Biolabs) was applied to insert the three resulting fragments into the suicide pRL271 vector linearized by SpeI. The resulting recombinant plasmid was conjugated into *Nostoc* as described above. The initial conjugants were selected by screening for resistance to 5 μg/mL of Sm, and the resulting cells were then grown on BG11 plates containing 5% sucrose to select double recombinants. Genomic DNA of the recombinant cells were analyzed by PCR.

The strains and plasmids used in this study are listed in Table 3.

**Table 3:** List of the bacterial strains and the plasmids used in this study

Strain name	Description/antibiotic resistance	Origin
Wild type	*Nostoc/Anabaena* PCC 7120 wild type strain	Pasteur Cyanobacterial Collection
WT/*patB*-*flv3B*	*Nostoc* containing the pRL25T-*patB*-*flv3B* plasmid/(Neo^R^)	This study
Δ*hupL*	*Nostoc* deletion mutant of the *hupL* gene (Sp/Sm^*R*^)/	This study
Δ*hupL*/*patB*-*flv3B*	Δ*hupL* mutant containing the pRL25T-*patB*-*flv3B*/(Sp/Sm^*R*^ and Neo^R^)	This study

Plasmid name	Description/antibiotic resistance	Origin
pRL25T	Replication vector derived from the pRL25C cosmid (Neo^R^)	[26, 30]
pRL25T-*patB*-*flv3B*	pRL25T harboring the *flv3B* gene under the control of the *patB* promoter (Neo^R^)	This study

### RNA preparation and reverse transcription

RNAs were prepared using the Qiagen RNA extraction kit (Qiagen) following the manufacturer instructions. An extra TURBO DNase (Invitrogen) digestion step was undergone to eliminate the contaminating DNA. The RNA quality was assessed by tape station system (Agilent). RNAs were quantified spectrophotometrically at 260 nm (NanoDrop 1000; Thermo Fisher Scientific). For cDNA synthesis, 1 µg total RNA and 0.5 μg random primers (Promega) were used with the GoScript™ Reverse transcriptase (Promega) according to the manufacturer instructions.

### Quantitative real-time-PCR for transcriptional analyses

Quantitative real-time PCR (qPCR) analyses were performed on a CFX96 Real-Time System (Bio-Rad). The reaction volume was 15 μL and the final concentration of each primer was 0.5 μM. The qPCR cycling parameters were 95 ℃ for 2 min, followed by 45 cycles of 95 ℃ for 5 s, 55 ℃ for 60 s. A final melting curve from 65 ℃ to 95 ℃ was added to determine the specificity of the amplification. To determine the amplification kinetics of each product, the fluorescence derived from the incorporation of BRYT Green^®^ Dye into the double-stranded PCR products was measured at the end of each cycle using the GoTaq^®^ qPCR Master Mix 2X Kit (Promega). The results were analysed using Bio-Rad CFX Maestro software, version 1.1 (Bio-Rad, France). The *rnpB* gene was used as a reference for normalization. A technical duplicate was performed for each point. The amplification efficiencies of each primer pairs were 80 to 100%. All of the primer pairs used for qPCR are reported in Table 2.

### Western blot analysis

Proteins (75 µg) extracted from cyanobacterial strains were fractionated by performing SDS-PAGE 12%, and transferred to nitrocellulose membranes before being revealed with specific polyclonal antibodies. Immune complexes were detected with anti-rabbit peroxidase-conjugated secondary antibodies (Promega) and enhanced chemoluminescence reagents (Pierce). Anti-FlvB antibodies, developed against the FlvB protein of *C. reinhardtii* [18], were used at a 1: 1000 dilution. Anti-Rbcl antibodies (Agrisera) were used a 1: 5000 dilution.

### H_2_ production assays

*Nostoc* wild type strain and its derivatives were grown as described above for heterocyte induction. Chlorophyll *a* concentration was quantified according to the following method: 1 mL of culture was centrifuged (5 min, 6700 g, 4 ℃), the pellet was resuspended in 1 mL of cold methanol and incubated at 4 ℃ for 30 min under shaking. Cells were then harvested (5 min, 6700 g, 4 ℃) and absorbance of the supernatant was measured at 665 nm and 720 nm. The chlorophyll *a* concentration was calculated according to the formula: [Chl a] = 12,9447 (A_665_–A_720_) and expressed in µg of Chla/mL of culture [28]. A 40-mL volume of cell culture was then harvested (5 min, 6700 g, 4 ℃) and cells were resuspended in sterile nitrate-depleted medium yielding a concentration of 10 μg Chl*a* mL^−1^. 12 mL of this cell suspension were transferred to Hungate tubes (leaving a 4.4-mL head space volume). The vials were sparged with Argon (Ar), and the samples were maintained under illumination (20 or 60 μmol photons m^−2^ s^−1^) for 96 h. 100 μL of headspace gas was removed every 12 h using a gastight syringe and injected into a gas chromatography system (Agilent 7820) equipped with a thermal conductivity detector and a HP-plot Molesieve capillary column (30 m, 0.53 mm, 25 µm), using argon as the carrier gas, at a flow rate of 4.2 mL/min, an oven temperature of 30 ℃ and a detector temperature of 150 ℃. H_2_ was quantified according to a standard calibration curve. H_2_ production rate was expressed as mol of H_2_ produced per mg of Chlorophyll.

### Nitrogenase activity

An on-line acetylene reduction assay [29] was used to measure nitrogenase activity. Briefly, cyanobacterial strains were grown in batch cultures under light/dark cycles of 12 h/12 h. Nitrogenase activity was monitored for 20 h. Before the onset of nitrogenase activity, *Nostoc* cultures were transferred to a GF/F filter (Whatman, 47 mm) and placed in a custom-made, light and temperature-controlled gas flow-through incubator connected to the gas chromatograph. Acetylene represented 10% of the gas mixture and the total gas flow rate was 1 l h^−1^. Ethylene production was measured every 10 min by gas chromatography using an Agilent 7890 equipped with an auto-injector and a photoionization detector.

## Supplementary information

**Additional file 1: Figure S1.** Alignment of the amino acid sequence of the Flv3B protein of *Nostoc* (all0178) and FlvB of *Chlamydomonas reinhardtii* (Cre16.g691800.t1.1).

## Data Availability

All the data supporting the conclusions of this article are included within the article and its additional file.
