# Supplementary figures and images for: Overproduction of the Flv3B flavodiiron, enhances the photobiological hydrogen production by the nitrogen-fixing cyanobacterium Nostoc PCC 7120
Source: Microb Cell Fact. 2020 Mar 10;19:65. doi: 10.1186/s12934-020-01320-5 (PMC7063810; doi:10.1186/s12934-020-01320-5)

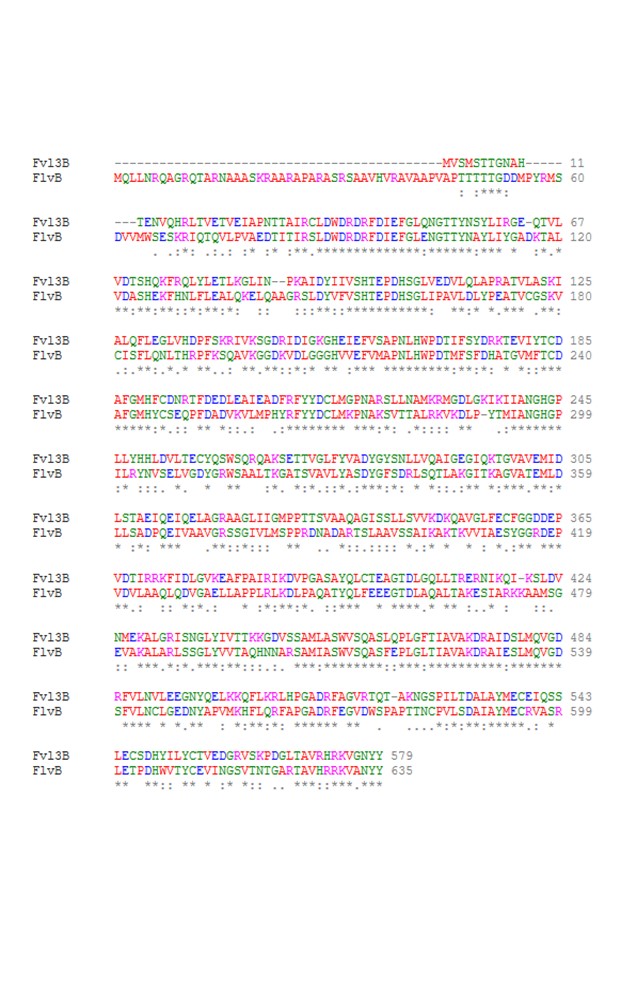

Supplement: Supplementary file 1 — Additional file 1: Figure S1. Alignment of the amino acid sequence of the Flv3B protein of Nostoc (all0178) and FlvB of Chlamydomonas reinhardtii (Cre16.g691800.t1.1). [file 12934_2020_1320_MOESM1_ESM.jpg]
